# Efficacy and safety of paclitaxel drug-coated balloon angioplasty for stenosis of hemodialysis vascular access: 6-month results from a brazilian multicenter prospective study

**DOI:** 10.1590/1677-5449.202401032

**Published:** 2025-02-14

**Authors:** Leonardo de Oliveira Harduin, Thiago Almeida Barroso, Julia Bandeira Guerra, Márcio Gomes Filippo, Leonardo Cortizo de Almeida, Carlos Alexandre Rosa Gama, Brunno Ribeiro Vieira, Renata Silveira Mello, Adriano Martins Galhardo, Jorge Paulo Strogoff-de-Matos

**Affiliations:** 1 Universidade do Estado do Rio de Janeiro – UERJ, Rio de Janeiro, RJ, Brasil.; 2 Complexo Hospitalar de Niterói - CHN/DASA, Niterói, RJ, Brasil.; 3 Centro Clínico LivCare, Niterói, RJ, Brasil.; 4 Colégio Brasileiro de Radiologia e Diagnóstico por Imagem – CBR, São Paulo, SP, Brasil.; 5 Universidade Federal do Rio de Janeiro – UFRJ, Rio de Janeiro, RJ, Brasil.; 6 Hospital Ana Nery, Salvador, BA, Brasil.; 7 Hospital São Luiz Anália Franco, São Paulo, SP, Brasil.; 8 Instituto Nacional de Traumatologia e Ortopedia, Rio de Janeiro, RJ, Brasil.; 9 Universidade Federal Fluminense – UFF, Niterói, RJ, Brasil.

**Keywords:** hemodialysis, paclitaxel-coated balloon, Ranger^TM^, vascular access, stenosis, hemodiálise, balão revestido com paclitaxel, Ranger™, acesso vascular, estenose

## Abstract

**Background:**

Stenosis resulting from neointimal hyperplasia remains a significant concern associated with dysfunction of arteriovenous fistulas (AVF).

**Objectives:**

To investigate the safety and efficacy of paclitaxel drug-coated balloon (DCB) angioplasty for treating failing AVFs.

**Methods:**

Investigators analyzed 58 hemodialysis patients treated with Ranger^TM^ DCBs from December 2022 to December 2023 across four centers. Lesions treated were de novo or restenotic and located in the juxta-anastomosis, cannulation zone, and outflow segment. Patients were evaluated through physical examinations and Duplex ultrasound at 1, 3, and 6 months. The primary efficacy endpoint was target lesion primary patency at 1, 3, and 6 months, and the primary safety endpoint was freedom from serious adverse events through 30 days post-procedure. Secondary endpoints were access circuit primary patency and technical and procedural success.

**Results:**

Nine patients (16%) had thrombosed access at the initial presentation, and 31 (53%) presented with recurrent stenosis. The target lesion primary patency rate at 6 months was 85.7%, and the access circuit primary patency rate at 6 months was 67.5%. No serious adverse events, either local or systemic, were reported. Sex, age, stenosis location, type of lesion, presence of thrombosis, lesion recurrence, diabetes status, or whether post-ballooning dilation was performed did not significantly affect the 6-month target lesion primary patency.

**Conclusions:**

DCB angioplasty was shown to be safe and effective for treating peripheral stenosis in vascular access.

## RESUMO

**Contexto:**

A estenose resultante da hiperplasia neointimal continua sendo uma preocupação significativa associada à disfunção das fístulas arteriovenosas (FAVs).

**Objetivos:**

Investigar a segurança e eficácia da angioplastia com balão revestido com paclitaxel (DCB) para o tratamento de FAVs em falência.

**Métodos:**

Os investigadores analisaram 58 pacientes em hemodiálise tratados com o DCB Ranger™ de dezembro de 2022 a dezembro de 2023, em quatro centros. As lesões tratadas eram *de novo* ou restenóticas, localizadas próximas à anastomose, na zona de canulação ou no segmento de saída. Os pacientes foram avaliados por meio de exames físicos e ultrassom Duplex aos 1, 3 e 6 meses. O desfecho primário de eficácia foi a perviedade primária da lesão-alvo aos 1, 3 e 6 meses, e o desfecho primário de segurança foi a ausência de eventos adversos graves até 30 dias após o procedimento. Os desfechos secundários foram a perviedade primária do circuito de acesso e o sucesso técnico e procedimental.

**Resultados:**

Nove pacientes (16%) apresentaram trombose das FAVs na apresentação inicial e 31 (53%) apresentaram estenose recorrente. A taxa de perviedade primária da lesão-alvo aos 6 meses foi de 85,7%, e a taxa de perviedade primária do circuito de acesso aos 6 meses foi de 67,5%. Nenhum evento adverso grave, local ou sistêmico, foi relatado. Sexo, idade, localização da estenose, tipo de lesão, presença de trombose, recorrência da lesão, *status* diabético e realização de dilatação pós-balão não afetaram significativamente a perviedade primária da lesão-alvo aos 6 meses.

**Conclusões:**

A angioplastia com DCB demonstrou ser segura e eficaz para o tratamento da estenose periférica em acessos vasculares.

## INTRODUCTION

Functional autologous arteriovenous fistulas (AVFs) are the vascular access of choice for patients with end-stage renal disease undergoing hemodialysis therapy. AVFs are favored over prosthetic arteriovenous grafts and central venous catheters due to their longer durability, fewer complications, and lower maintenance costs.^1-4^ However, stenosis resulting from neointimal hyperplasia remains a major concern associated with AVF dysfunction.^5^ Additionally, about 50% of AVFs fail to mature due to development of intimal hyperplasia and stenosis, leading to reduced flow and access occlusion.^5-7^

Plain balloon angioplasty, typically with high-pressure balloons, remains the first-line treatment for clinically significant stenosis.^1^ Despite being widely used, the technique’s primary patency rates at one year are relatively low, ranging from 26% to 62%, mainly due to cellular proliferation and neointimal hyperplasia that occur post-procedure.^5,8,9^ Addressing recurrent AVF dysfunction often requires repeated plain balloon angioplasties, a costly approach that can also negatively impact patient quality of life.^8-10^

Recent studies have shown that angioplasty using drug-coated balloons offers better primary patency rates than traditional plain balloon angioplasty.^11-13^ These drug-coated balloons are typically infused with paclitaxel, an agent that stabilizes microtubules and prevents migration and proliferation of vascular smooth muscle cells, which are key factors in the development of neointimal hyperplasia.^14^

This study reports the 6-month results from a prospective, single-arm trial investigating the safety and efficacy of DCB angioplasty for treating failing autologous AVFs.

## MATERIAL AND METHODS

### Ethical aspects

All procedures in this study followed the ethical standards of the 1964 Helsinki Declaration and its later amendments. The research ethics committee at the Universidade Federal Fluminense, Rio de Janeiro, Brazil, approved this study under opinion number 5.747.190 and certificate of presentation of ethical appreciation number 55923222.1.0000.5243. Patients provided written informed consent to participate in the study, and their personal details were removed from this analysis.

### Patient selection

The investigators prospectively analyzed a convenience sample of patients with AVF treated for stenosis of hemodialysis vascular access using the Ranger™ DCB (Boston Scientific, Marlborough, MA, USA) between December 2022 and December 2023. The study was conducted at four centers: 1) Centro Clínico Livcare (Niterói, Rio de Janeiro, Brazil), 2) Clínica Vene (Rio de Janeiro, Rio de Janeiro, Brazil), 3) Clínica Inteligência Vascular Avançada (Salvador, Bahia, Brazil) and 4) Dermavasc (Brasília, Distrito Federal, Brazil). The sample size was determined by the availability of patients who met the inclusion criteria within the study period.

Patients included in the study underwent angioplasty for salvage of AVF access and met the following inclusion criteria: they were aged over 18 years; had an autologous AVF in use for at least 30 days; exhibited significant angiographic stenosis (luminal narrowing ≥ 50%), causing clinical and hemodynamical dysfunction of the vascular access; had stenosis located between the arteriovenous anastomosis and the axillary vein; had a target lesion length of less than 10 cm, and a vessel diameter of less than 8 mm.

The exclusion criteria for the study included: pregnant or breastfeeding women; those planning to become pregnant during the study period; individuals with active infections; patients on immunosuppressive therapy; participation in other research studies; cognitive limitations preventing understanding of the nature of the study and its potential consequences; patients with coagulation disorders; life expectancy of less than 12 months; allergies to contrast media, aspirin, clopidogrel, heparin, or paclitaxel; and concurrent central venous stenosis.

### Study device

The Ranger™ (Boston Scientific, Marlborough, MA, USA) is a drug-coated balloon with a 2 μg/mm^2^ paclitaxel dose and a TRANSPAX™ citrate ester coating that transfers the drug while reducing paclitaxel exposure during catheter delivery. Once at the lesion site, paclitaxel irreversibly binds to microtubules, inhibiting proliferation of smooth muscle cells and reducing the occurrence of neointimal hyperplasia.^14^ The components of the drug-coated balloon are built on the platform of the commercially available Sterling (Boston Scientific, Marlborough, MA, USA) balloon dilation catheter, which is compatible with a 0.018-inch wire.

### Study procedures

Four vascular surgeons performed the procedures with patients under local anesthesia and sedation. A prophylactic dose of cephalosporin (1000 mg) was administered intravenously before the procedure. Following an initial fistulogram and intravenous administration of 5,000 IU of heparin, the stenosis was crossed using a 0.018 hydrophilic guidewire and pre-dilated with a high-pressure non-compliant balloon. The type and size of this balloon (chosen by the operator) were selected to match the diameter of the adjacent normal vessel. Multiple inflations were used for resistant lesions. If these lesions did not respond adequately to dilation, they were treated with a cutting or ultra-high pressure non-compliant balloon at the operator’s discretion. This treatment approach was also considered acceptable for inclusion in this study. Patients who needed a stent after the initial dilation due to indicators of vessel preparation failure, such as more than 30% residual stenosis, a dissection that limited blood flow, or leakage of contrast material, were excluded from the study.

Once good vessel preparation had been achieved, treatment was completed with inflation of a DCB (Ranger™, Boston Scientific, Marlborough, MA, USA) over the wire. To avoid geographic miss, the DCB was 1 mm larger in diameter and at least 5mm longer at either end than the last balloon dilatation catheter used. The Ranger™ drug-coated balloons had diameters of 5-8 mm and lengths of 40-80mm and were always inflated at their nominal pressure for 3 minutes. After completing DCB angioplasty, the operator could perform additional dilation with a high-pressure balloon to achieve satisfactory results, at their own discretion.

A final angiogram of the entire vascular access was performed to evaluate the procedural result, exclude any immediate complications, and serve as a reference for subsequent follow-up angiograms. Physical examinations and Duplex ultrasound were scheduled at 1, 3, and 6 months after the procedure, according to the routine protocol of the centers, with extra visits as needed to address access circuit dysfunction. Anticoagulants or antiplatelet therapy were used according to the standard care protocols at each center. Variables collected from the electronic records included patient demographics, comorbidities, procedural details, and outcome data.

### Study endpoints

The primary and secondary study endpoints are described in Table 1. We applied study definitions according to the Society of Interventional Radiology (SIR) reporting standards.^15^

**Table 1:** Study endpoints.

Endpoint type	Endpoint description	Endpoint definition
Primary endpoint	Target lesion primary patency at 1, 3, and 6 months	The time between intervention treatment and any subsequent intervention within the affected region
Primary safety endpoint	30-day safety	Freedom from localized or systemic serious adverse events through 30 days post-procedure
Secondary endpoint	Technical success	Uncomplicated angioplasty and less than 30% remaining stenosis
Secondary endpoint	Procedural success	The ability to perform a regular dialysis immediately after treatment
Secondary endpoint	Access circuit primary patency at 1, 3, and 6 months	The time spanning from DCB treatment to the initial intervention anywhere within the access circuit or an event of access circuit thrombosis

### Statistical analysis

Measured values are reported as frequencies. Patency analysis was performed with the Kaplan-Meier method and, when applicable, compared using the log-rank test. SPSS software version 18.0 for Windows (IBM, USA) was used for data analysis, and p-values < 0.05 were considered statistically significant. In this study, statistical power calculations were performed a posteriori to evaluate sample size adequacy for detecting clinically meaningful differences.

## RESULTS

Fifty-eight patients fulfilled the inclusion criteria and were evaluated in this study.

### Patient and lesion characteristics

Table 2 details patient demographics, concurrent medical conditions, and characteristics of the dialysis access circuits.

**Table 2:** Patient demographics, concurrent medical conditions, and characteristics of the dialysis access circuits (n=58).

Variable		N	%
Age	<65 years	31	53
	≥65 years	27	47
Sex	Male	33	57
	Female	25	43
AVF type	Radiocephalic	28	48
	Brachiocephalic	12	21
	Brachiobasilic	18	31
Target lesion location	Juxta-anastomosis	30	52
	Cannulation zone	10	17
	Outflow segment	18	31
Thrombosis	No	49	84
	Yes	9	16
Hypertension	No	9	16
	Yes	49	84
Diabetes mellitus	No	32	55
	Yes	26	45
Ischemic heart disease	No	48	83
	Yes	10	17
Smoker	No	52	90
	Yes	6	10
Stroke	No	54	93
	Yes	4	7
DCB diameter	5 mm	0	0
	6 mm	3	5
	7 mm	26	45
	8 mm	29	50
DCB length	40 mm	13	22
	60 mm	23	40
	80 mm	22	38
Post ballooning dilation	No	16	28
	Yes	42	72
Restenosis	De novo lesion	27	47
	Recurrent/restenotic lesion	31	53

AVF: arteriovenous fistula; DCB: paclitaxel drug-coated balloon.

Most patients were male (57%), under 65 years old (53%), and non-smokers (90%). Significant comorbidities included hypertension (84%), diabetes mellitus (45%), ischemic heart disease (17%), and stroke (7%). Fifty-three percent of patients presented with recurrent stenosis, and 47% with de novo lesions. Stenotic lesions were located in the juxta-anastomosis (52%), cannulation zone (17%), and outflow segment (31%). The types of AVF treated were radiocephalic (in 48% of patients), brachiocephalic (21%), and brachiobasilic (31%).

### Clinical, procedural, and safety outcomes

**Figure 1:**
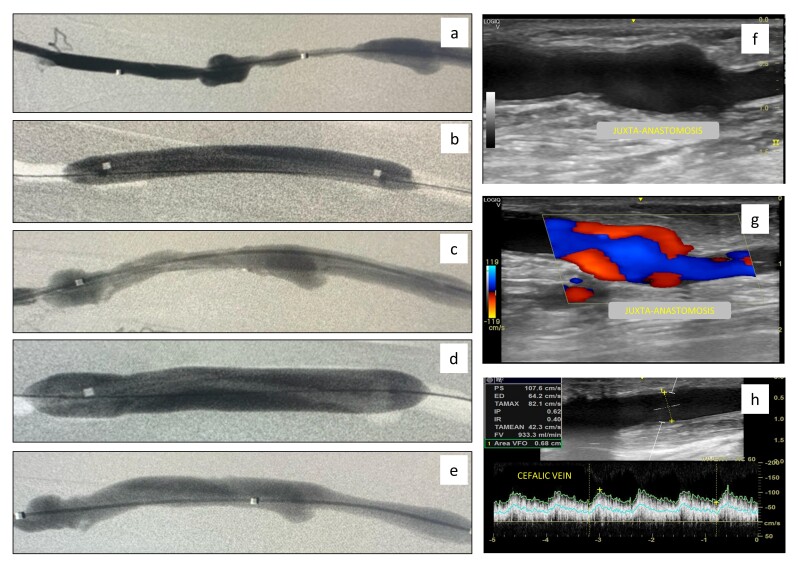
Example of procedural outcomes in a patient with radiocephalic AVF with stenosis at juxta-anastomosis. **a)** Angiography of a radiocephalic AVF with stenosis at juxta-anastomosis. **b)** Pre-dilation with 6x60mm high-pressure balloon; **c)** Preoperative angiography demonstrating adequate vessel preparation; **d)** Angioplasty with Ranger^TM^ DCB (8x80mm); **e)** Control angiography; **f, g,** and **h)** Control Doppler 6 months after the initial procedure without signs of restenosis and with a flow volume of 933ml/min.

Clinical and procedural success were achieved in 100% of the patients. Figure 1 shows the procedural outcomes of an example case. Throughout the first 30 days following the procedure, no serious adverse events, either local or systemic, were reported. No deaths occurred during the study period.

### Performance outcomes

**Figure 2:**
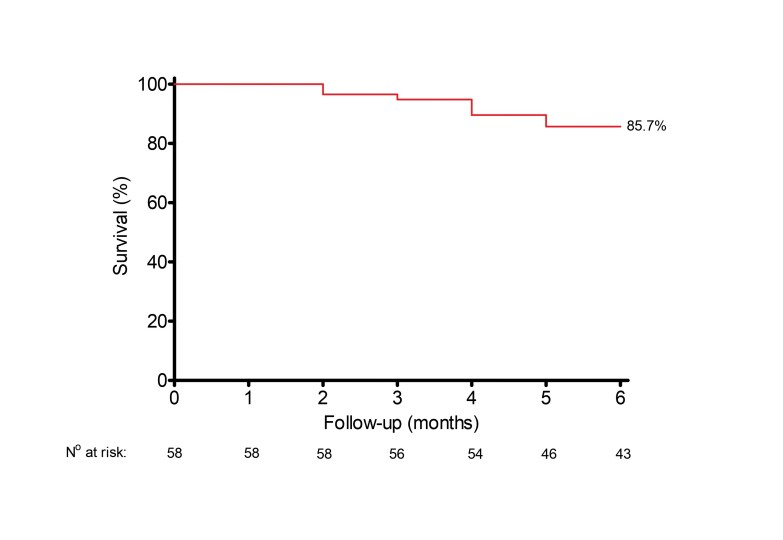
Target lesion primary patency rates were 100% at 1 month, 96.5% at 3 months, and 85.7% at 6 months.

**Figure 3:**
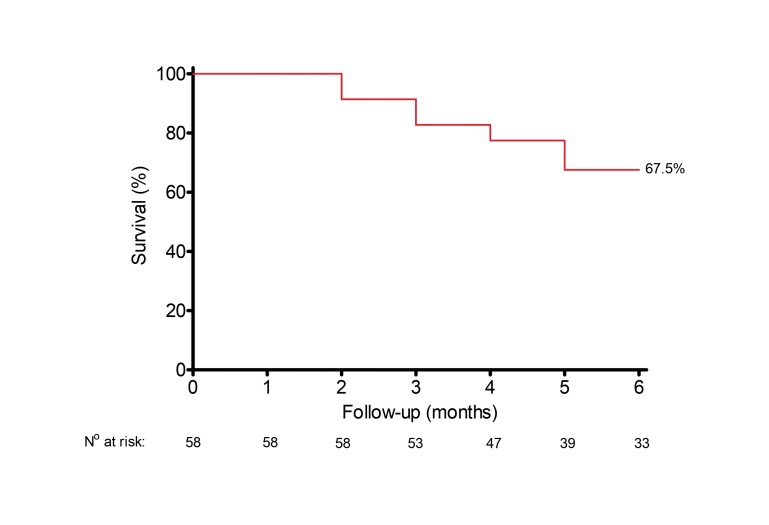
Access circuit primary patency rates were 100% at 1 month, 91.3% at 3 months, and 67.5% at 6 months.

Figures 2 and 3 show patency rates for the study endpoints over the 6-month follow-up period. As shown in Figure 2, target lesion primary patency rates at 1, 3, and 6 months were 100%, 96.5%, and 85.7%, respectively. Access circuit primary patency rates were 100% at 1 month, 91.3% at 3 months, and 67.5% at 6 months (Figure 3).

As detailed in Table 3, we did not observe significant differences in 6-month target lesion primary patency when comparing groups based on sex, age, stenosis location, type of lesion, presence of thrombosis, lesion recurrence, diabetes status, or post-ballooning dilation.

**Table 3:** Comparative analysis of six-month target lesion primary patency (TLPP) across patient demographics, lesion characteristics, and treatment variables (n=58).

Variable						TLPP	p-value
Age	<65 years					83.6%	0.58
	≥65 years					87.9%	
Sex	Male					90.2%	0.25
	Female					79.8%	
Target lesion location	Cannulation zone					88.9%	0.90
	Juxta-anastomosis					86.5	
	Outflow segment					83.0%	
Thrombosis	No					82.9%	0.19
	Yes					100%	
Diabetes mellitus	No					81.1%	0.24
	Yes					91.2%	
Post ballooning dilation	No					87.5%	0.57
	Yes					81.3%	
Recurrent lesion	No					91.7%	0.22
	Yes					80.5%	

## DISCUSSION

Fistula failure in patients relying on hemodialysis results in missed or inadequate dialysis treatments, repeated endovascular treatments, and catheter use, all adversely affecting patients’ quality of life and contributing to healthcare costs.^16^ Current research indicates that drug-coated balloons are safe and may reduce restenosis rates compared to conventional angioplasty balloons when treating failing AVFs.^17^

Building on this knowledge, our study demonstrates the effectiveness of DCB angioplasty for treating hemodialysis vascular access stenosis. We achieved a 100% clinical and procedural success rate in the patient cohort examined. Furthermore, the absence of deaths or serious adverse events related to the procedure within the 6 months following treatment indicates the safety of DCB angioplasty in dysfunctional dialysis fistulas.

The demographics and lesion characteristics of the patients in our cohort reflect a typical end-stage renal disease population, with a high prevalence of hypertension, diabetes, and recurrent stenosis.^18-20^ The distribution of stenotic lesions and types of AVFs treated is comparable to what has been reported in previous research, underscoring the common challenges in managing AVF stenoses.^20,21^

The 6-month target lesion primary patency rate of 85.7% observed in this study is notably high, particularly compared to the outcomes of traditional percutaneous transluminal angioplasty. A systematic review and meta-analysis of six randomized controlled trials and four cohort studies showed that target lesion primary patency in DCBs varied from 18.75% to 88.24% at 6-month follow-up.^22^ For lesions treated with non-drug coated balloons, rates ranged from 30% to 77.42%.^22^ This is the second study investigating the efficacy and safety of Ranger™ DCB angioplasty for hemodialysis vascular access stenosis. Previously, Soon et al.^23^ conducted a retrospective study comparing patency rates after the use of Ranger™ DCB or conventional balloon angioplasty in Asian patients with hemodialysis access stenosis. The study reported a higher, though not statistically significant, target lesion primary patency rate in the DCB-treated arm at 6 months (84.3% vs 71.6%, p=0.08), along with significantly longer mean time to target lesion reintervention, especially amongst recurrent lesions when compared to the conventional balloon angioplasty-treated arm. Additionally, DCB-treated circuits demonstrated a longer mean time to circuit reintervention (6.9 ± 2.8 vs 5.8 ± 3.7 months, p = 0.04). Also important is that the target lesion primary patency rate of the Ranger™ DCB in the Asian population^23^ closely matches the results we observed in the Brazilian population, highlighting the device’s consistent performance across diverse ethnic groups.

Paclitaxel is the most commonly used drug in drug-eluting balloons because it is highly lipophilic and rapidly absorbed and retained in endothelial cells after a short contact time.^24^ However, the technology behind DCBs comes at a higher cost compared to traditional or high-pressure balloons, limiting their use primarily to challenging lesions prone to recurrence.^17^ Our study showed comparable patency rates for de novo and restenotic lesions treated with DCBs, suggesting that broadening the clinical application of these devices beyond recurrent cases could be beneficial in reducing the overall need for repeat interventions. In the present study, other classical risk predictors such as sex, age, stenosis location, type of lesion, presence of thrombosis, diabetes status, and post-ballooning dilation did not statistically modify target lesion primary patency.

Our study presents some limitations, mainly the small number of participants and the relatively short follow-up period. Considering the sample size of 58 patients, the present study would only have an adequate statistical power of 80% if the absolute difference in patency rate between subgroups were 30% or higher. To better evaluate the difference in the effectiveness of DCB angioplasty for specific settings, it would be necessary to include more participants and/or extend the follow-up period. Additionally, the use of a convenience sample may not provide a representative cross-section of the broader population due to potential biases in patient availability. Consequently, the results should be interpreted with caution, acknowledging that they may not fully extrapolate to all individuals with hemodialysis vascular access stenosis.

The call for randomized clinical trials to more definitively determine the efficacy of Ranger™ DCBs across different lesion types and patient characteristics is a crucial next step. Randomized clinical trials would provide a controlled environment to isolate the effects of DCBs, mitigating the influence of confounding variables and offering stronger evidence to support or refute our preliminary observations. Larger observational studies would also provide more robust data and allow for a more granular analysis of the efficacy and safety of DCB angioplasty across different patient demographics and lesion characteristics. Such studies would help clarify the specific indications for DCB use, optimizing patient selection and treatment outcomes.

We plan to analyze the 12-month outcomes in this patient population to better understand the long-term effectiveness and safety of angioplasty with the Ranger™ DCB in treating hemodialysis vascular access stenosis. This extended follow-up will provide additional information on the durability of the treatment effect, the potential for late adverse events, and the need for subsequent interventions, which are crucial for establishing long-term treatment protocols.

## CONCLUSION

Our study demonstrates the effectiveness and safety of angioplasty with the Ranger™ DCB for treating hemodialysis vascular access stenosis. We achieved high clinical and procedural success rates with no significant adverse events within the 6-month follow-up period. The 6-month target lesion primary patency rate of 85.7% suggests that DCB angioplasty is a valuable addition to the treatment options for failing AVFs. Future studies, particularly randomized controlled trials and larger observational studies, are essential to refine patient selection criteria and optimize treatment protocols.
